# Acute kidney injury induces high-sensitivity troponin measurement changes after cardiac surgery

**DOI:** 10.1186/s12871-017-0307-5

**Published:** 2017-01-31

**Authors:** Amr S Omar, Khaled Mahmoud, Samy Hanoura, Hany Osman, Praveen Sivadasan, Suraj Sudarsanan, Yasser Shouman, Rajvir Singh, Abdulaziz AlKhulaifi

**Affiliations:** 1 Department of Cardiothoracic Surgery/Cardiac Anaesthesia and ICU, Heart Hospital, Hamad Medical Corporation, Doha, PO 3050, Qatar; 2 Department of Critical Care Medicine, Beni Suef University, Beni Suef, Egypt; 3 Weill Cornell Medical College-Qatar, Doha, Qatar; 4 Department of Nephrology, Hamad Medical Corporation, Doha, PO 3050, Qatar; 5 Department of Nephrology, Faculty of Medicine, Mansoura University, Mansoura, Egypt; 6 Department of Anesthesia, Al-Azhar University, Cairo, Egypt; 7 Department of Cardiology Research Center, Hamad Medical Corporation, Doha, Qatar

**Keywords:** Acute kidney injury, High sensitive troponin, Cardiac surgery

## Abstract

**Background:**

The value of cardiac troponin as a risk assessment tool for cardiac disease in the setting of end-stage renal diseases (ESRD) is not equivalent to its value in those with normal renal function. This consideration had not been studied in settings of acute kidney injury (AKI). We aim to explore the diagnostic value of high sensitive troponin T (hsTnT) in the settings of cardiac surgery-induced AKI.

**Methods:**

Single center observational retrospective study. Based on the AKI Network, patients divided into 2 groups, group I without AKI (259 patients) and group II with AKI (100 patients) where serial testing of hsTnT and creatine kinase (CK)-MB were followed in both groups. Patients with (ESRD) were excluded.

**Results:**

The mean age in our study was 55.1 ± 10.2 years. High association of AKI (27.8%) was found in our patients. Both groups were matched regarding the age, gender, body mass index, the association of diabetes or hypertension, and Euro score. AKI group had significantly higher mortality 5% vs group I 1.1% (*p* = 0.03). The hsTnt showed a significant sustained rise in the AKI group as compared to the non-AKI group, however CK-MB changes were significant initially but not sustained.

The AKI group was more associated with heart failure 17.9% vs 4.9% (*p* = 0.001); and post-operative atrial fibrillation, 12.4% vs 2.9% (*p* = 0.005). Lengths of ventilation, stays in ICU and in hospital were significantly higher in the AKI group.

**Conclusions:**

Unlike the CK-MB profile, the hsTnT showed significant changes between both groups all over the course denoting possible delayed clearance in patients with AKI.

## Background

The ultimate role of cardiac biomarkers in cardiac surgery remains unresolved, but such biomarkers are currently used in international guidelines to monitor and define myocardial infarction. The ideal analytical infrastructure and target proteins should be highly sensitive for early and late diagnosis, highly specific, easy to use, and affordable. These proteins should improve patient outcomes and impact therapeutic modalities [1]. Three subunits form the backbone of the troponin complex. The C, I and T subunits have been studied previously and are located on the myofibrillar thin (actin) filament of striated (skeletal and cardiac) muscle. Cardiac muscle expresses the isoforms troponin T and I, and cardiac troponin T (cTnT) and I (cTnI) are more specific than creatine kinase (CK) values for myocardial injury. Thus, these proteins may be elevated when CK-MB is not [2].

The risk assessment of cardiac troponin and other cardiac biomarkers in end-stage renal disease and normal renal function is not equivalent. Clinical decision making based on cardiac biomarkers in patients with renal diseases requires justification in relation to patient management or outcomes [3].

A wide variety of causes may be associated with increases in cardiac enzymes after surgery, including acute coronary syndrome (ACS) related to recent acute myocardial infarction (AMI) before surgery [4] or perioperative myocardial infarction (PMI) directly related to cardiac surgery. These enzymes may also be elevated due to incomplete cardio-protection, reperfusion injury, and direct surgical trauma [5]. Elevation during cardiac surgery may not be ACS-related because such elevations may be caused by end-stage renal disease [6], acute pericarditis [7], acute heart failure (AHF), sepsis [8] and rhabdomyolysis [9].

PMI has a variable incidence and is reported after coronary artery bypass surgery (CABG) [10]. This phenomenon occurs both on and off pump, as well as in valvular surgeries, although it is relatively unusual. The reported incidence is up to 5% after CABG and 4% after valvular surgeries [11, 12].

The recently introduced high-sensitivity troponin T (hsTnT) showed higher sensitivity and negative predictive values than conventional cTn [13]. This marker exhibits excellent diagnostic performance, even with early presentation to the emergency department in the AMI scenario [14]. There is some evidence that hsTnT may possess better diagnostic accuracy than cTn [15]. hsTnT was introduced in 2010 and has been available at Hamad Hospital since June 2011. This protein not only serves as a sensitive and rapid diagnostic tool but may also aid in short- and long-term mortality prediction.

Cardiac troponin is related to short- and long-term mortality in the setting of cardiac surgery [16]. PMI is diagnosed based on a CK-MB increase of more than 10-fold (99th percentile upper reference level) in combination with electrocardiogram (ECG), echocardiographic or angiographic evidence [17]. Acute kidney injury (AKI) is a frequent complication after cardiac surgery, with a reported incidence of up to 30%. Renal insufficiency and acute cerebral events were associated with hsTnT elevation in the emergency department in a cross-sectional study performed by Lindner et al. [18].

No previous studies highlighted the influence of AKI on cardiac hsTnT. In a recent study performed by Prowle and Kirwan, the authors reported that long-term outcomes could be influenced after AKI in cardiac surgery, but cardiac troponins must be studied in these settings [19]. We hypothesize that hsTnT could be subjected to changes in the settings of AKI after cardiac surgery.

### Aim of the work

Our goal was to explore the diagnostic value of hsTnT in the setting of cardiac surgery-induced AKI (with or without cardiac events).

## Methods

1. Study design and assessments
  This study was a single-center retrospective observational study that was conducted over 18 months (March 2011 to September 2013) in a 12-bed cardiothoracic ICU at Hamad Medical Corporation in Doha, Qatar. The ethical committee approved the study (reference number 15068/15), and written informed consent was waived for all subjects. We excluded patients with sepsis, preexisting high levels of hsTnT, end-stage renal disease (ESRD), and AKI before the surgery, as well as patients with marked intraoperative hypotension (mean arterial blood pressure less than 33% of the initial value for more than 10 min) [20]. A total of 19 patients were excluded. We recruited 359 consecutive patients who underwent cardiac surgery. The sample size was calculated to be a minimum of 320 subjects [21].
  The following data were recorded for all patients: age, sex, existence of diabetes or hypertension, surgery type, length of anesthesia, cardiopulmonary bypass (CPB) time, aortic cross clamp (ACC) time, use of vasopressors and inotropes, and Euro SCORE. Outcome variables, including the length of mechanical ventilation (LOV), the length of stay in the ICU (LOS_ICU_) and the length of stay in the hospital (LOS_hosp_), were recorded. Routine liver and renal functions were recorded. Complications, including AKI, post-operative atrial fibrillation (POAF), infection, stroke, and in-hospital mortality, were recorded for each patient. Dendrite Clinical Systems (London, UK) was used to retrieve data. Blood samples for hsTnT and CK-MB measurements were collected every six hours for the first 24 h after surgery and according to clinical indications after the first 24 h. hsTnT was measured using COBAS Troponin T hs (highly sensitive) on a STAT (short turn-around time) instrument (Roche Diagnostics). ECGs were performed routinely before and immediately after surgery and every 12 h after surgery. Transthoracic echocardiography was performed for all patients before surgery and was then requested when indicated to trace new regional wall motion abnormalities in patients with high levels of cardiac enzymes and in patients requiring high doses of inotropes.

2. Outcome definitions
  The primary outcome was association of hsTnT with post cardiac surgery AKI, the later was defined according to the consensus definition proposed by the Acute Kidney Injury Network. Here, AKI was defined as an abrupt (within 48 h) reduction in kidney function, which was defined as an absolute increase in the serum creatinine concentration of 0.3 mg/dL or greater (26.4 μmol/L) or a percentage increase of 50% or greater (1.5-fold from baseline) [22]. The secondary outcome measures were the length of mechanical ventilation (LOV), the LOS_ICU_, and the length of hospitalization. The patients were divided into two groups based on AKI status: Group I experienced no AKI, and Group II experienced AKI.

3. Normally distributed continuous variables were expressed as the mean ± standard deviation (SD). Skewed variables were presented as the median (interquartile range (IQR). The patients were divided into two groups based on the existence of AKI. Continuous variables were compared using the Student’s *t*-test and the Mann Whitney *U* test, as appropriate. Chi-square or Fisher’s exact tests were used to compare categorical variables between the two groups. A significant association was defined by a probability (P) value ≤ 0.05 (two-tailed). HsTnT was converted to natural logarithm values for the purpose of multivariate linear regression analysis. Linear multivariate analysis putting the outcome variables was done in relation to HsTnT, Statistical analysis was performed using the SPSS software (version 22, Chicago, IL, USA).

## Results

Of the 378 patients screened, 359 patients were enrolled in the study; the remaining 19 patients met one or more of the exclusion criteria. Males were predominant in the study population of 303 patients (84.9%). The mean age was 57.2 ± 11.6 years, and the majority of patients underwent CABG surgery (291 patients (82.6%); Tables 1 and 2).

**Table 1:** Description of the studied group

Variable	Number	Minimum	Maximum	Mean ± SD
Age	359	18	75	57.2 ± 11.6
BMI (kg/m^2^)	358	17.1	46.9	28.1 ± 7.1
Creatinine (micromole/L)	359	54.1	178.7	97.1 ± 60.1
EF%	355	20	66	50.1 ± 10.1
Additive Euro score	354	0	18	3.6 ± 2.9
CPB time (minutes)	356	0	344	120.7 ± 56.3
ACC time (minutes)	356	0	180	86.1 ± 40.1
Anesthesia time (minutes)	355	200	648	301 ± 81
LOV (minutes)	354	210	4701	502 ± 481
LOS_hosp_ (days)	354	6	499	35.1 ± 30.6

*BMI* body mass index, *EF* ejection fraction, *CPB* cardiopulmonary bypass, *ACC* aortic cross clamp, *WBCs* white blood cells, *LOV* length of mechanical ventilation, *LOS*_*hosp*_ Hospital length of stay

**Table 2:** Demographic differences between both groups

Variable	Group I (No AKI) 259 (%)	Group II (AKI) 100 (%)	*P*- Value
Age	54.43 ± 10.8	56.09 ± 10.7	0.13
Gender (male)	222 (85.7)	83 (83)	0.34
Diabetes	138 (53.2)	56 (56)	0.38
Hypertension	124 (47.8)	59 (59)	0.13
Euro score	3.8 ± 2.4	5.1 ± 3.6	0.06
BMI	28.1 ± 5.8	28.9 ± 6.2	0.12
EF < 40	14 (5.4)	21 (21)	0.001
Elective surgery	165 (63.7)	57 (57)	0.065
Basal creatinine (micromole/L)	88.2 ± 41.7	92.1 ± 42.3	0.6
Surgery			
CABG	212 (82.4)	79 (79)	0.6
Valvular	30 (16)	18 (18)	0.7
Aortic disssection	4 (1.3)	1 (1)	

*BMI* body mass index, *EF* ejection fraction, *CABG* coronary artery bypass graft

The patients were divided into two groups based on the existence of AKI. Group I consisted of patients with no AKI, and Group II consisted of patients with AKI (Tables 2 and 3). The two groups were well matched in terms of age, gender, hypertension, additive Euro score, body mass index (BMI), baseline creatinine and surgery type. A low ejection fraction (EF) was more notable in the AKI group (Table 2). The AKI group had significantly higher requirements for inotropes and vasopressors. There were no significant differences between the groups in terms of anesthesia time; however, CPB and ACC were significantly higher in the AKI group (Table 3).

**Table 3:** Main differences in both studied groups

Variable	Group I (No AKI) 259 (%)	Group II (AKI) 100 (%)	P- Value
Inotrops			
Dopamine	19 (7.3)	11 (11)	0.05
Adrenaline	12 (4.6)	14 (14)	0.05
Noradrenline	34 (13.1)	24 (24)	0.03
Dobutamine	2 (0.9)	2 (2)	0.7
Intraoperative parameters			
CPB time (minutes)	119 ± 43	135 ± 69.6	0.06
ACC time (minutes)	77.1 ± 33	77.4 ± 48.5	0.09
Anesthesia time (minutes)	6.8 ± 1.4	6.7 ± 2	0.9
Postoperative parameters			
LOV(minutes)	364.1 ± 112	575.5 ± 199	0.001
LOS_ICU_ (hours)	52.9 ± 41.1	109.4 ± 89	0.000
LOS_hosp_ (days)	10.1 ± 3.4	16.7 ± 4.7	0.001
Post-Operative outcome			
POAF	7 (2.7)	12 (12)	0.005
Inhospital-mortality	3 (1.1)	5 (5)	0.03
VAP	5 (1.9)	2 (2)	0.8
Re-admission ICU	8 (3)	6 (6)	0.07
Re-exploration	24(6.5)	11 (26.8)	0.02
PMI	2 (0.8)	6 (6)	0.02

*AKI* acute kidney injury, *CPB* cardiopulmonary bypass, *ACC* aortic cross clamp, *LOV* length of mechanical ventilation, *LOS*_*ICU*_ ICU length of stay, *LOS*_*hosp*_ Hospital length of stay, *POAF* post-operative atrial fibrillation, *VAP* ventilator associated pneumonia

The outcome variables, including the lengths of ventilation, ICU and hospital stay, were significantly higher in the AKI group. Post-operative complications, including post-operative atrial fibrillation (POAF), hospital-mortality and re-exploration, were significantly more frequent in the AKI group. No significant differences in the incidence of VAP and re-admission to the ICU were observed between groups. PMI was more frequent in patients with PMI (Table 3). Finally, Table 4 illustrates the relation of the outcome variables to HsTnT values.

**Table 4:** Outcome variables in relation to HsTnT

	Beta coefficient	Significance
AKI	.47	0.001
peri op MI	.91	0.013
LOS_HOSP_ (days)	.005	0.090
LOV (hours)	.004	0.001
POAF	.82	0.06
Mortality	1.1	0.2
CONSTANT	6.81	0.001

*AKI* acute kidney injury, *PMI* perioperative myocardial infarction, *LOS*_*hosp*_ Hospital length of stay, *LOV* length of mechanical ventilation, *POAF* post-operative atrial fibrillation

Linear multivariate analysis for troponin values

**Fig. 1:**
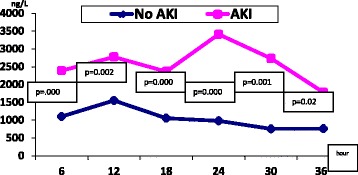
Changes in hsTnT in both groups

**Fig. 2:**
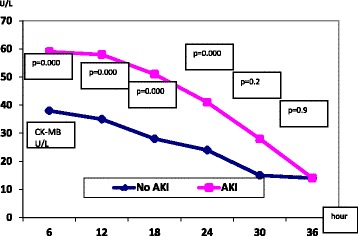
Changes in CK-MB in both groups

Significant differences in hsTnT were observed between the two groups (Fig. 1) throughout the study period. The same changes were associated with CK-MB early in the study period but not later in the study period (Fig. 2).

## Discussion

The salient findings of this study were as follows: 1) AKI patients exhibit changes in hsTnT but not CK-MB after cardiac surgery injury; 2) high mortality and morbidity were observed in the group with AKI in terms of POAF events and re-exploration; and 3) the AKI group exhibited prolonged lengths of ventilation, ICU and hospital stay. The utility of sensitive cardiac troponin for the early diagnosis of acute cardiac events has already been demonstrated [14]. The predictive value of cardiac troponin for patient outcomes in ESRD has been reported for a long time [5]. In addition, the incidence of AKI and the potential impact on patients undergoing cardiac surgery was elucidated in some previous studies [19]. Meanwhile, the impact of AKI on cardiac troponins and their implications after cardiac surgery have not yet been studied.

To our knowledge, this study is the first to evaluate the diagnostic performance of hsTnT in the setting of cardiac surgery-induced AKI. This study showed that hsTnT may be an inaccurate marker of perioperative myocardial infarction in those patients who develop AKI post cardiac surgery. We showed that hsTnT is raised in AKI patients independent of cardiac events. While a few studies have addressed troponin in AKI [23], we are not aware of any studies that investigated hsTnT.

Rosner and Okusa reported previously that the pathogenesis of AKI associated with cardiac surgery involves multiple pathways. These pathways include inflammatory, hemodynamic, and nephrotoxic factors and may include other factors that are involved and overlap with each other, leading to kidney injury [24]. Risk factors for AKI have been identified in some clinical trials, and these factors can be used to determine the risk for AKI in patients who undergo cardiac surgery. The major risk factors include diabetes mellitus, hypertension, female gender, heart failure and a low EF [24].

In our study cohort, we encountered AKI in 27.8% of patients, which is within the range reported in the literature [24]. However, we believe that this value is relatively high. This high percentage could be attributed to the high incidence of diabetes mellitus, hypertension and low EF in the study group. These factors would expose the patients to a higher risk of developing AKI.

Acute kidney injury is strongly associated with poor outcomes in terms of morbidity and mortality—especially in the setting of cardiac surgery [24, 25]. In our study, mortality and other outcome measures were significantly higher in the AKI group than the non-AKI group. Most outcome parameters were significantly higher in the AKI group. The requirement for more inotropic support, the length of ventilation (LOV), the LOS_ICU_ and the LOS_Hosp_ were significantly elevated. These findings are consistent with other clinical reports in which high mortality and more complicated hospital courses were encountered in patients who underwent cardiac surgery and developed AKI.

Mao et al. performed a prospective, multicenter cohort study of 1,219 patients and confirmed the relationship with important clinical outcomes, including a longer length of hospital and ICU stay and a higher risk of dialysis or death [25]. However, the occurrence of AKI could be a sensitive indicator of the severity of the perioperative insult and poor physiological reserves, as well as a direct cause of the poor outcome [19]. Similarly we found changes in the outcome variables with higher levels of HsTnT (Table 4).

In patients with chronic kidney disease, it is possible to have elevated cardiac troponins even in the absence of true cardiac injury. This elevation could occur due to different processes, including decreased clearance and possible re-expression of cardiac troponin in uremic myopathic skeletal muscles [2]. Meanwhile, several studies have reported that increased cardiac troponin in end-stage renal disease patients is an independent predictor of both short- and long-term mortality [5, 26].

In addition, elevated cardiac troponin levels in AKI patients post-cardiac surgery result in different effects, including myocardial cell injury due to incomplete cardioprotection, direct surgical trauma and possible reperfusion injury. This observation does not reflect perioperative myocardial infarction [2]. Here, we found that significant POAF events were associated with the AKI group. This association was also reported in a recent study by Kristovic et al. [27].

In our study cohort, we demonstrated a persistent rise in cardiac hsTnT in the AKI group across the entire time course. This pattern was not observed for the CK-MB marker. This trend of rising hsTnT in the setting of AKI should be considered when evaluating the diagnostic performance of hsTnT in patients with recent cardiac surgery for a cardiac insult—especially in the absence of a clear hsTnT diagnostic discrimination point. Omar et al. defined values (3,466 ng/L and 2,309 ng/L) for hsTnT that accompany PMI and myocardial injury, respectively [28]. We found that PMI events were significantly more frequent in patients with AKI (Table 3), PMI was associated with high levels of HsTnT (Table 4). Perhaps when AKI develops after cardiac surgery, the biochemical diagnosis of a new cardiac insult using cardiac hsTnT should be based on a rising trend rather than a parallel rise or a defined value while keeping in mind the expected rise in hsTnT throughout the AKI course.

Study strengths, limitations and recommendations:

To our knowledge, this study is the first study to describe hsTnT changes in the setting of AKI. However, the findings should be interpreted with some caution. We recognize some limitations of our study, such as the short follow-up period, the retrospective design, and the lack of cut-off values for CK-MB and hsTnT, as well as the lack of a correlation with creatinine levels. The study was conducted in one center, and long-term follow up of the patients was impossible due to the highly dynamic work conditions in Qatar. These limitations could be further addressed in future studies.

## Conclusion

Unlike the CK-MB assay, hsTnT exhibited significant changes in the AKI group, suggesting possible delayed clearance. A rise in hsTnT should be interpreted cautiously as a marker of post-operative myocardial injury and infarction in patients with AKI.

## Abbreviation

ACC: Aortic cross clamp
AKI: Acute kidney injury
AMI: Acute myocardial infarction
CK-MB: Creatine kinase MB
CPP: Cardiopulmonary bypass time
EF: Ejection fraction
ESRD: End stage renal disease
hsTntT: High sensitive troponin T
ICU: Intenzsive care unit
LOS_Hosp_: Length of stay in hospital stay
LOS_ICU_: Length of stay in ICU
LOV: Length of ventilation
PMI: Perioperative myocardial infarction
POAF: Post-operative atrial fibrillation
